# Symmetrical awareness network for cross-site ultrasound thyroid nodule segmentation

**DOI:** 10.3389/fpubh.2023.1055815

**Published:** 2023-03-08

**Authors:** Wenxuan Ma, Xiaopeng Li, Lian Zou, Cien Fan, Meng Wu

**Affiliations:** ^1^Electronic Information School, Wuhan University, Wuhan, China; ^2^Department of Ultrasound, Zhongnan Hospital of Wuhan University, Wuhan, China

**Keywords:** thyroid nodule segmentation, thyroid nodule classification, domain adaptation, ultrasound image processing, medical image segmentation

## Abstract

Recent years have seen remarkable progress of learning-based methods on Ultrasound Thyroid Nodules segmentation. However, with very limited annotations, the multi-site training data from different domains makes the task remain challenging. Due to domain shift, the existing methods cannot be well generalized to the out-of-set data, which limits the practical application of deep learning in the field of medical imaging. In this work, we propose an effective domain adaptation framework which consists of a bidirectional image translation module and two symmetrical image segmentation modules. The framework improves the generalization ability of deep neural networks in medical image segmentation. The image translation module conducts the mutual conversion between the source domain and the target domain, while the symmetrical image segmentation modules perform image segmentation tasks in both domains. Besides, we utilize adversarial constraint to further bridge the domain gap in feature space. Meanwhile, a consistency loss is also utilized to make the training process more stable and efficient. Experiments on a multi-site ultrasound thyroid nodule dataset achieve 96.22% for PA and 87.06% for DSC in average, demonstrating that our method performs competitively in cross-domain generalization ability with state-of-the-art segmentation methods.

## 1. Introduction

According to global Cancer statistics 2020 (1), thyroid cancer has become one of the fastest-growing cancers in the past 20 years, ranking in 9th place for incidence. Early symptoms manifest as thyroid nodules, and then as the disease progresses, patients gradually feel pain. If not promptly detected and treated in the early stage, thyroid cancer can cause significant harm to patients and even be life-threatening. Therefore, early and accurate assessment is of crucial importance for improving the chances of cure and survival for patients.

In thyroid diagnosis, ultrasound imaging technique (2) has become the preferred imaging modality due to many advantages such as convenience, good reproducibility, and low cost (3, 4). Usually, radiologists diagnose patients base on the ultrasound characteristics of the images, which requires physicians to have rich experience and superb technology. With the increasing number of thyroid patients year by year, the current demand for radiologists is increasing so fast that it is no longer sufficient to rely on manual diagnosis to meet the needs of society.

Since machine learning and deep learning (5) pervade medical image computing, they are becoming increasingly critical in the medical imaging field, including medical image segmentation (6–12). Leveraging learning-based techniques, multiple novel methods have been proposed to conduct medical image segmentation tasks. Compared with traditional mathematical morphology-based methods, learning-based ones have achieved impressive results. In practice, however, there remain several challenges for deep learning-based methods. One salient problem is that deep learning lacks generalization capability, resulting in models trained on data from one site can not achieve good results on the data from other sites. This is because the ultrasound images from different sites show discrepancies in appearance and contrast due to different imaging protocols, examination equipment and patient groups, which is called domain shift. Due to the existence of domain shift, the model obtained only through deep learning methods cannot be adapted to different sites. In addition, since it is difficult to label medical data, most medical centers still maintain a state where it is difficult to use deep neural network algorithms.

Domain adaptation (13) is a valid approach to address the domain shift problem. The core task of domain adaptation is to tackle the differences in probability distribution between the source domain and the target domain by learning robust knowledge. Existing researches on domain adaptation for medical imaging can be divided into two categories. The first category aims at the feature transfer (14). It learns transferable and discriminative features across different domains (15, 16). Specifically, it maps features from source and target domains to the same distribution *via* certain transforms (17). The second category aims at the model transfer (17). It learns transferable models by fine-tuning or other methods in the target domain (18, 19). One widely-used example is transferring parameters from the pre-trained model on ImageNet (20) to other tasks. Those two categories can greatly improve the generalization capacity of deep learning-based model by dealing with the domain heterogeneity. However, these methods still suffer from certain limitations. First, many methods inevitably require a few labeled target data for fine-tuning. This restricts their performance to unsupervised scenarios. Meanwhile, medical image annotations often require considerable efforts and time. Second, as for model transfer, dataset bias (21) deteriorates the transferring performance. Namely, when the source domain, e.g., ImageNet, differs too much from the target domain, e.g., medical images, this method achieves only average performance. Third, these methods only perform monodirectional domain shift, namely, source to target. Therefore, the image translation functions may lead to undesirable distortions.

In this paper, we propose a domain adaptation framework for medical image segmentation. Our architecture is composed of image translation module and image segmentation module. Medical images from different domains have different styles at the pixel level, and the rule also applies to our source and target domain. Inspired by Cyclegan (22), we use the image translation module to realize the translation between the source domain and the target domain, and guide the process with a pixel-level adversarial loss. Apart from pixel-level alignment, the alignment of semantic features also has a great impact on image segmentation tasks. Therefore, we further unify the style of latent vectors drawn from the segmentation network, and guide the process with a feature-level adversarial loss. The domain gap is well bridged through two-step alignment on both the pixel and feature level. Our segmentation module is constructed into two symmetrical parts to realize the task of segmentation in both the source and target domain, respectively. In each branch, we utilize Efficientnet (23), with strong feature extraction capabilities, to extract deep semantic features and build an image segmentation network based on the encoder-decoder structure. In order to enhance the feature fusion ability and improve the segmentation performance, we use the hybrid channel attention mechanism to concat the features between the encoder and the decoder. Ultimately, considering that the segmentation results from the two branches of the same image should be consistent, we introduce the segmentation consistency loss to further guide the unlabeled branch in an unsupervised manner. In short, our main contributions and novelty of the paper could be summarized as follows:

(1) We propose a domain adaptation framework for medical image segmentation which can narrow the domain gap between different data and effectively improve the generalization ability.
(2) We apply multi-level domain adaptation to simultaneously bridge the domain gap on both pixel-level and feature-level through adversarial learning, and obtain better adaptation results.
(3) Considering the invariance of the segmentation results of the same target in the domain adaptation process, we implement bidirectional symmetric awareness through segmentation consistency loss to further improve the stability and performance of our model.

### 1.1. Related works

#### 1.1.1. Medical image segmentation

To tackle the medical image segmentation problem, traditional segmentation methods focus on the contour, shape and region properties of thyroid nodules (24–28), while mainstream researchers now focus more on deep learning-based methods. Wang et al. (6) apply multi-instance learning and attention mechanism to automatically detect thyroid nodules in three stages, the feature extraction network, the iterative selection algorithm, and the attention-based feature aggregation network. Peng et al. (7) propose an architecture that combines low-level and high-level features to generate new features with richer information for improving the performance of medical image segmentation. Zhang et al. (8) propose a multiscale mask region-based network to detect lung tumors, which trains multiple models and acquires the final results through weighted voting. Tong et al. (9) propose a novel generative adversarial network-based architecture to segment head and neck cancer. This method uses the shape representation loss and 3D convolutional autoencoder to strengthen the shape consistency of predictions of the segmentation network. Similarly, Trullo et al. (10) propose to use distance-aware adversarial networks to segment multiple organs. This method leverages the global localization information of each organ along with the spatial relationship between them to conduct the task. Li et al. (11) utilize the widely-anticipated transformer to process the medical image. This method applies squeezed and expanded attention blocks to encode and decode features extracted from CNN. Also, inspired by transformer, Cao et al. (12) propose to conduct image segmentation using a modified transformer-based architecture to improve the performance by combining global branch and local one.

#### 1.1.2. Domain adaptation for medical image analysis

As a promising solution to tackle domain heterogeneity among multi-site medical imaging datasets, domain adaptation has attracted adequate attention in the field. He et al. (29) propose to conduct the domain shift procedure using adversarial network. This method uses a label predictor and a domain discriminator to draw the domain distance closer. Li et al. (18) propose a modified subspace alignment method to diminish the disparity among different datasets, which aligns the sample points from separate feature spaces into the same subspace. Zhang et al. (30) propose the task driven generative adversarial networks to transfer CT images to X-ray images by leveraging a modified cycle-GAN sub-structure with add-on segmentation supervisions to learn the transferable knowledge. Chen et al. (31) propose an unsupervised domain adaptation framework, utilizing synergistic learning-based method to conduct domain translation from MR to CT. Ahn et al. (32) propose an unsupervised feature augmentation method. In this method, image features extracted from a pre-trained CNN are augmented by proportionally combining the feature representation of other similar images. Yoon et al. (33) propose to mitigate dataset bias by extending the classification and contrastive semantic alignment (CCSA) loss that aims to learn domain-invariant features. Dou et al. (34) propose to tackle the domain shift by aligning the feature spaces of source and target domains by utilizing the plug-and-play adaptation mechanism and adversarial learning. Perone et al. (35) propose to conduct domain adaptation in semi-supervised scenarios. Containing teacher models and student models, this method leverages the self-ensembling mechanism to improve the generalization of the models. Gao et al. (36) propose a lesion scale matching approach to use latent space search for bounding box size to resize the source domain images and then match the lesion scales between the two disease domains by utilizing the Monte Carlo Expectation Maximization algorithm. Kang et al. (37) propose intra- and inter-task consistent learning, where task inconsistency is restricted, to have a better performance on all tasks like thyroid nodule segmentation and classification. Gong et al. (38) design a thyroid region prior guided feature enhancement network (TRFEplus) for the purpose of utilizing prior knowledge of thyroid gland segmentation to improve the performance of thyroid nodule segmentation.

## 2. Materials and methods

### 2.1. Data acquisition

Our ultrasound thyroid nodule datasets consist of three domain data collected from different patients in different medical centers with different ultrasound systems. The first two datasets are private datasets, which contain 936 and 740 images, respectively, while the third dataset is the public dataset DDTI (39) containing 637 images.

### 2.2. Method overview

**Figure 1:**
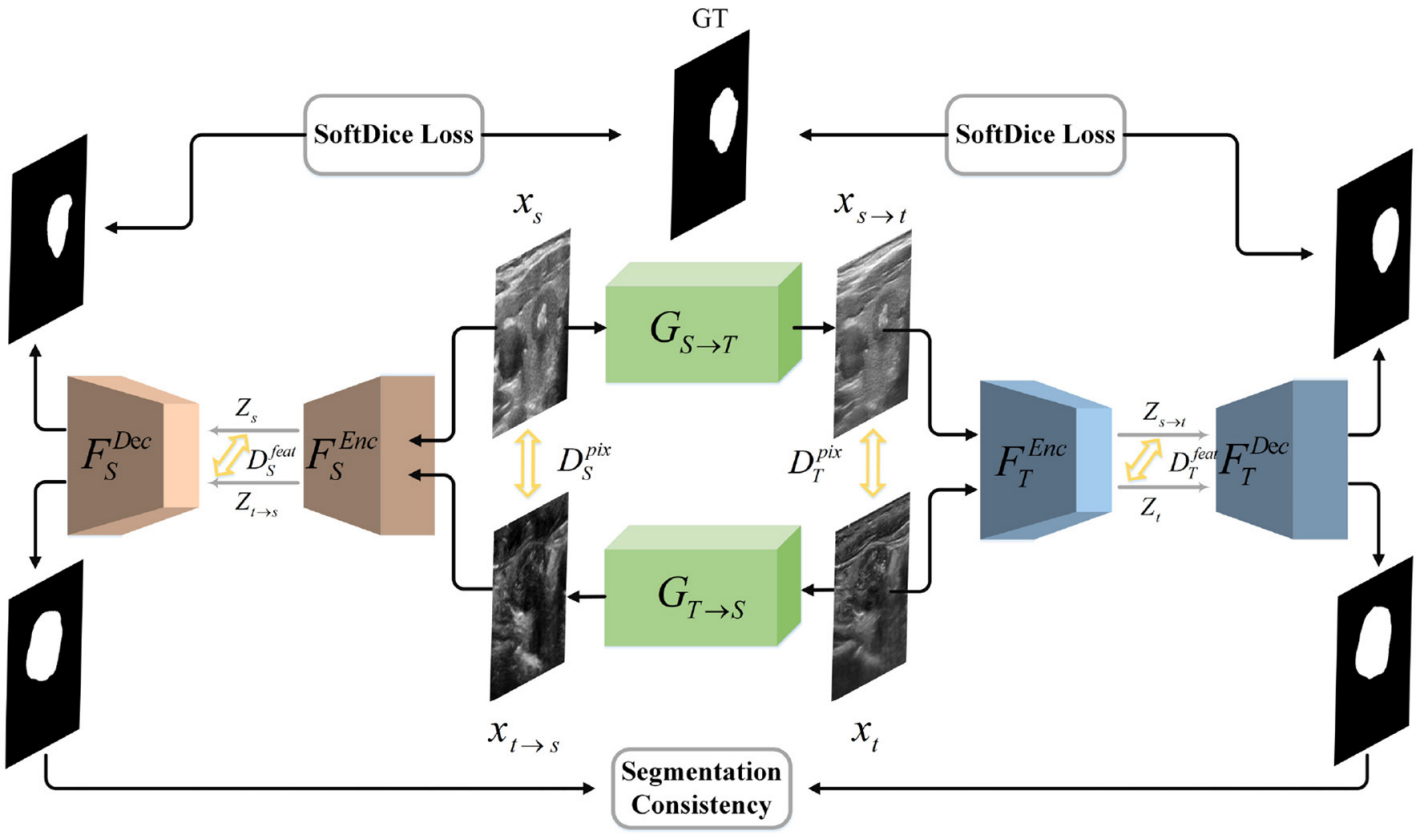
Illustration of our network architecture. We design a bidirectional translation module to respectively generate *x*_*t*→*s*_ and *x*_*s*→*t*_. We introduce adversarial loss to unify the style of translated and target domain on both the pixel and feature level. Then we utilize two symmetrical segmentation modules, i.e., *F*_*S*_ and *F*_*T*_, to respectively achieve segmentation in two domains. SoftDice loss and segmentation consistency loss are proposed to further conduct the network.

In this work, we aim to build a segmentation network with remarkable cross-domain generalization ability. Specifically, given a labeled dataset $X_{S}={\left\{x_{s}\right\}}_{s=1}^{N_{S}}$ in source domain and an unlabeled dataset $X_{T}={\left\{x_{t}\right\}}_{r=1}^{N_{T}}$ in target domain, where *N*_*S*_ and *N*_*T*_ denote the number of images, we assume that they obey the marginal distributions *P*_*S*_(*x*_*s*_) and *P*_*T*_(*x*_*t*_). The domain adaptation problem can be defined as mapping *X*_*S*_ and *X*_*T*_ to corresponding latent spaces *via* $F_{S}^{Enc}:X_{S}\to Z_{S}$, $F_{T}^{Enc}:X_{T}\to Z_{T}$, respectively. The representations *Z*_*S*_ and *Z*_*T*_ are desired to obey the same distribution, so that *Z*_*S*_≈*Z*_*T*_. Consequently, we present a novel bidirectional symmetric segmentation framework, as is shown in Figure 1. Designed to close the domain gap on both the pixel level and feature level, our framework is divided into one bidirectional image translation module and two symmetric image segmentation modules. At the pixel level, we introduce the image translation module, i.e., *G*_*S*→*T*_ and *G*_*T*→*S*_, where *G*_*S*→*T*_ translates images from source domain to target domain while *G*_*T*→*S*_ performs image translation inversely. Considering of the fact that semantic information has a more profound impact on image segmentation, we propose to unify the style of latent vectors drawn from the segmentation network on the feature level. Given *x*_*t*_ and *x*_*s*→*t*_, the segmentation module is utilized to encode them into latent codes *z*_*t*_ and *z*_*s*→*t*_. And an adversarial discriminator is utilized to close their domain gap, encouraging them to obey the same distribution. Consistent with the bidirectional translation module, we introduce two symmetrical segmentation branches, i.e., *F*_*S*_ and *F*_*T*_, to respectively achieve segmentation in the source and target images. In each branch, the Efficientnet (23) and the hybrid channel attention mechanism are introduced to enhance the feature extraction and fusion capability of our segmentation modules. Besides, the segmentation results of *x*_*t*_ and *x*_*t*→*s*_ should be the same because they represent the same content information. The rule also applies to the relationship between *x*_*s*_ and *x*_*s*→*t*_. Therefore, we introduce the segmentation consistency loss to further guide the network.

### 2.3. Network architecture

#### 2.3.1. Translation module

**Figure 2:**
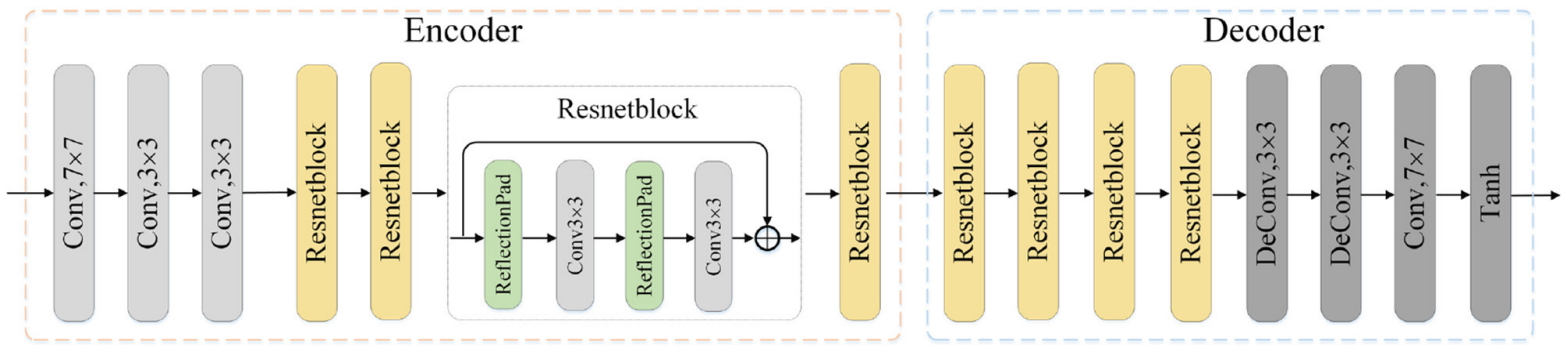
Illustration of one translation module architecture. The input is the image *x* of source or target domain and the output is the translated image. Conv: convolution layer, Deconv: deconvolution layer, Tanh: Hyperbolic Tangent.

The image translation modules are designed to close the domain gap on both the pixel and feature level. Each module consists of one encoder network and the corresponding decoder network. As is shown in Figure 2, the source image is first fed into the encoder to generate the latent codes, which is then decoded into generated image by the decoder. The process also applies to the translation of target image. Besides, the 8-layer residual blocks are utilized to improve the network's learning ability. The encoder consists of one convolution block mapping image to high-dimension space, two downsampling convolution blocks with stride 2 and residual blocks. In terms of the decoder, we utilize two trainable deconvolution layers instead of the traditional upsampling blocks to improve the translation performance.

#### 2.3.2. Segmentation module

**Figure 3:**
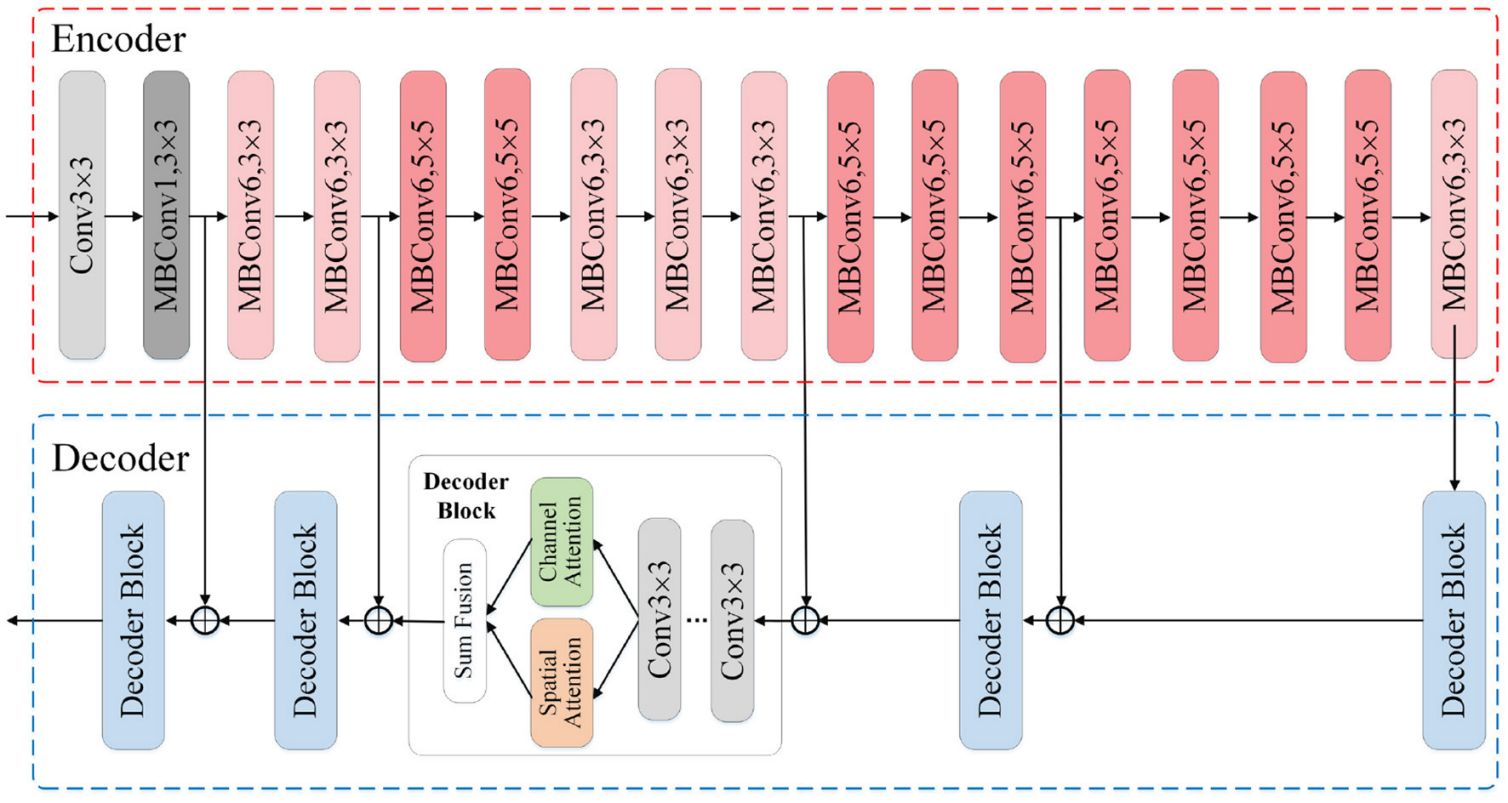
Illustration of the segmentation module architecture. The input is the original images or the ones translated by image translation module, and the output is the segmentation result. The encoder adopts EfficientNet-B0 as the feature extraction skeleton. In the decoder we incorporate the hybrid attention mechanism and skip connection mechanism.

Based on the image segmentation framework UNet, our segmentation modules adopt the EfficientNet-B0 as feature extraction network, also the hybrid attention mechanism to enhance the expression ability of fusion feature and further improve the segmentation performance. The architecture of our segmentation modules is shown in Figure 3. We construct the modules with an encoder-decoder architecture, where the encoder is utilized to extract multi-scale feature maps while the decoder translates the low-resolution feature maps back into images of original size. The EfficientNet-B0 module in the encoder mainly uses the Mobile Inverted Bottleneck convolution layer to extract features related to the target, and alternately uses the MBConv modules with different convolution kernel sizes to expand the receptive field. To make full use of the location information contained in the shallow features, we integrate the skip connection mechanism into the encoder-decoder architecture to fuse the shallow features from encoder and the deep features from decoder. After that, we feed the fusion feature into the channel attention module and the spatial attention module, respectively, to obtain the feature maps whose channel and spatial semantic information are calibrated. By adding the two calibrated feature maps, new features with global dependence come into being.

### 2.4. Loss functions

#### 2.4.1. Image translation loss

As is discussed above, we utilize the image translation modules to bridge the domain gap on both the pixel and feature level. The discriminators incorporated denote two feature-level discriminators ($D_{S}^{feat}$ and $D_{T}^{feat}$), and two pixel-level discriminators ($D_{S}^{pix}$ and $D_{T}^{pix}$). Take the source domain discriminators for example, the $D_{S}^{feat}$ is adopted to narrow the gap between the feature map of the source image *x*_*s*_ and the translated image *x*_*t*→*s*_, while the $D_{S}^{pix}$ is adopted to close the gap on the pixel level. The adversarial losses of source domain are shown as follows.


(1) $$\begin{array}{l} {ℒ}_{S}^{\text{adv }}=E\left[-\log\left(1-D_{S}^{\text{feat }}\left(x_{t\to s}\right)\right)\right]+E\left[-\log\left(D_{S}^{\text{feat }}\left(x_{s}\right)\right)\right]\\ \;+E\left[-\log\left(1-D_{S}^{pix}\left(x_{t\to s}\right)\right)\right]+E\left[-\log\left(D_{S}^{pix}\left(x_{s}\right)\right)\right]. \end{array}$$

Similar to the source domain adversarial losses, the adversarial losses of target domain are shown as follows.


(2) $$\begin{array}{l} {ℒ}_{T}^{\text{adv }}=E\left[-\log\left(1-D_{T}^{\text{feat }}\left(x_{s\to t}\right)\right)\right]+E\left[-\log\left(D_{T}^{\text{feat }}\left(x_{t}\right)\right)\right]\\ \;+E\left[-\log\left(1-D_{T}^{pix}\left(x_{s\to t}\right)\right)\right]+E\left[-\log\left(D_{T}^{pix}\left(x_{t}\right)\right)\right]. \end{array}$$

Besides, we introduce the cycle-consistency loss (22) to further conduct the translation module. Explicitly, if we feed the image *x*_*s*_ to *G*_*S*→*T*_ and then to *G*_*T*→*S*_, the result obtained should be the same as the original image *x*_*s*_. The rule also applies to the image *x*_*t*_. The cycle-consistency loss is shown as follows.


(3) $${ℒ}^{\text{cycle }}=E\left[{\left\|G_{T\to S}\left(x_{s\to t}\right)-x_{s}\right\|}_{1}\right]+E\left[{\left\|G_{S\to T}\left(x_{t\to s}\right)-x_{t}\right\|}_{1}\right].$$

Moreover, we also adopt an identity mapping loss (22) to prevent the generators from producing undesired results. For instance, the result of feeding the image *x*_*s*_ to *G*_*T*→*S*_ should be indistinguishable from the original input *x*_*s*_. The loss is shown as follows.


(4) $${ℒ}^{\text{iden }}=E\left[{\left\|G_{T\to S}\left(x_{s}\right)-x_{s}\right\|}_{1}\right]+E\left[{\left\|G_{S\to T}\left(x_{t}\right)-x_{t}\right\|}_{1}\right].$$

#### 2.4.2. Segmentation loss

The segmentation modules are divided into two parts to realize image segmentation in the source and target domain, respectively. Since the source domain image *x*_*s*_ is labeled, we train the segmentation process of *x*_*s*_ and *x*_*s*→*t*_ in a supervised manner. We utilize Dice loss as the supervised loss, which is defined as follows.


(5) $${ℒ}^{\mathrm{seg}}={ℒ}_{DICE}\left({ℱ}_{S}\left(x_{s}\right),\text{GT}\right)+{ℒ}_{DICE}\left({ℱ}_{T}\left(G_{S\to T}\left(x_{s}\right)\right),\text{GT}\right).$$

In terms of the target domain image *x*_*t*_ which is unlabeled, we train its segmentation process in an unsupervised manner and propose a consistency loss, which is shown as follows.


(6) $${ℒ}^{\text{consis }}={ℒ}_{DICE}\left({ℱ}_{T}\left(x_{t}\right),{ℱ}_{S}\left(G_{T\to S}\left(x_{t}\right)\right)\right).$$

Our consistency loss aims at guide the unlabeled branch with the supervised networks in labeled branch.

The overall loss function is defined as follows:


(7) $$\begin{array}{l} L=\lambda_{adv}\left({ℒ}_{S}^{adv}+{ℒ}_{T}^{\text{adv }}\right)+\lambda_{\text{cycle }}{ℒ}^{\text{cycle }}+\lambda_{\text{iden }}{ℒ}^{\text{iden }}+\lambda_{\text{seg }}{ℒ}^{\text{seg }}\\ \;+\lambda_{\text{consis }}{ℒ}^{\text{consis }}, \end{array}$$

Where the hyper-parameters λ_*adv*_, λ_*cycle*_, λ_*iden*_, λ_*seg*_, λ_*consis*_ denote the weight of each term.

### 2.5. Metrics

In the task of medical image segmentation, each pixel in the image can be divided into *Positive*, which means that exact area belongs to Thyroid Nodule, and *Negative* with the opposite meaning. For any image segmentation method, there would be *TruePositive*, *TrueNegative*, *FalsePositive* and *FalseNegative* representing 4 types of relationship between the result and the ground truth, denoted by *TP*, *TN*, *FP*, *FN*. To verify how well our method can tackle the medical image segmentation problem, the following evaluating metrics are utilized.

Pixel Accuracy(*PA*): The most commonly utilized metric in image segmentation task. It can be seen as the accuracy in pixel level, defined as the percentage of the correctly predicted pixels in total pixels. Formally, *PA* is defined as follows.


(8) $$\begin{array}{l} PA=\frac{TP+TN}{TP+FP+FN+TN}. \end{array}$$

Dice Similarity Coefficient(*DSC*) (40): Another wildly utilized metric in image segmentation problems. It can be used to evaluate the similarity of two groups, which represent predict result and ground truth in this situation. Formally, *DSC* is defined as follows.


(9) $$\begin{array}{l} DSC=\frac{2TP}{FP+2TP+FN}. \end{array}$$

True Positive Rate(*TPR*): Defined as the percentage of the correctly predicted pixels of positives in total positive pixels given by ground truth. In this case it can represent the ability of detecting positive area, thus known as Sensitivity. Formally, *TPR* is defined as follows.


(10) $$\begin{array}{l} TPR=\frac{TP}{TP+FN}. \end{array}$$

True Negative Rate(*TNR*): Defined as the percentage of the correctly predicted pixels of negatives in total negative pixels given by ground truth. In this case it can represent the ability of not being confused by negative area, thus known as Specificity. Formally, *TNR* is defined as follows.


(11) $$\begin{array}{l} TNR=\frac{TN}{TN+FP}. \end{array}$$

## 3. Experimental results and discussion

### 3.1. Implementation

#### 3.1.1. Data processing

Considering that each image frame contains unnecessary text which would affect the image segmentation performance, we only preserve the part with content information and remove the remaining. After that, we unify the size of the cropped image to 400*400. From each dataset, we randomly choose 200 images as the test set, others as the train set.

#### 3.1.2. Training details

During the training process, in order to ensure that our network can work effectively, we optimize our network step by step. The whole process is divided into three steps. Firstly, the image translation module is trained with adversarial loss to get optimized *G*_*S*→*T*_ and *G*_*T*→*S*_. Then given the inputs *x*_*s*_ and *G*_*S*→*T*_(*x*_*s*_), we train source domain segmentation network *F*_*S*_ and target domain segmentation network *F*_*T*_, respectively in a supervised manner. Finally, under the constraint of the total loss *L*, we carry out a more refined optimization of the whole network. In the training process, we set λ_*adv*_ = λ_*cycle*_ = λ_*iden*_ = 1, λ_*seg*_ = 10, λ_*consis*_ = 2. The whole experiment is carried out with four 1080Ti.

#### 3.1.3. Comparison methods

We compare our network with the following state-of-the-art methods: DeepLabV3 (41), PSPNet (42), FPN, PAN (43), and TRFEplus (38).

### 3.2. Ultrasound thyroid nodule segmentation

We carry out the experiment of ultrasound thyroid nodule segmentation to evaluate our proposed method. Three ultrasound thyroid nodule datasets with annotations are labeled as domain1, domain2, and domain3, respectively. Each time we select two of them, one of which is used as the source domain and the other is used as the target domain, then 6 sets of experiments are conducted.

We compare our method with several state-of-the-art methods using the four evaluating metrics above to compare the performance. For all the compared methods, we carry out comprehensive data augmentation to improve their generalization ability. Besides the commonly used geometric transformations, we also introduce noise, blur, occlusion, etc. to improve their performance as much as possible. Meanwhile, we exclude any data augmentation strategy in our method and only employ the domain adaptation architecture. For our method, we conduct two sets of experiment, one of which is applying data augmentation to our segmentation module only (namely SegM+AUG), and the other is our proposed domain adaptation framework. It should be noted that we exclude the data augmentation strategy from the latter scheme because the data augmentation will change the style of images and thus influence the translation process on pixel level.

Utilizing the metrics mentioned in Section 2.5, the results are presented in Tables 1–7. As can be seen from the tables, our method performs more favorably against other methods, especially in the most representative metric *DSC*, which confirms the feasibility of our domain adaptation method. Specifically, our method increases the average *PA* by 0.99%, the average *DSC* by 3.64% and the average *TNR* by 0.24%. It is worth noting that if the confusing pixels are judged as negative ones mostly, the *FP* will be greatly reduced, and the *FN* will be greatly increased as a cost. That is to say, this strategy improves the *TPR* by sacrificing *TNR*, which makes the results of TRFEplus (38) in Table 5 close to ours on *TNR* but far behind ours on *TPR*. From Tables 6, 7, we can see that the performance of the network applied domain adaptation is better than the one with data augmentation strategy.

**Table 1:** Results on ultrasound thyroid nodule datasets with DeepLabV3 (41) +AUG.

**Source**	**Target**	**PA**	**DSC**	**TPR**	**TNR**
Domain1	Domain2	0.9505	0.8461	0.9186	0.9531
Domain1	Domain3	0.9494	0.8579	0.9450	0.9477
Domain2	Domain1	0.8348	0.6991	0.9962	0.8003
Domain2	Domain3	0.7858	0.5444	0.9910	0.7596
Domain3	Domain1	0.9681	0.9071	0.8638	0.9939
Domain3	Domain2	0.9669	0.8623	0.8298	0.9892
Average		0.9093	0.7862	**0.9241**	0.9073

Bold values mean that the value is the best among all the comparison methods and our method.

**Table 2:** Results on ultrasound thyroid nodule datasets with PSPNet (42) +AUG.

**Source**	**Target**	**PA**	**DSC**	**TPR**	**TNR**
Domain1	Domain2	0.8581	0.6287	0.9336	0.8474
Domain1	Domain3	0.7955	0.5766	0.9220	0.7801
Domain2	Domain1	0.7835	0.6215	0.9610	0.7460
Domain2	Domain3	0.7263	0.4673	0.9582	0.6974
Domain3	Domain1	0.8740	0.9249	0.8903	0.9942
Domain3	Domain2	0.8902	0.3878	0.3037	0.9952
Average		0.8213	0.5678	0.8281	0.8434

**Table 3:** Results on ultrasound thyroid nodule datasets with FPN +AUG.

**Source**	**Target**	**PA**	**DSC**	**TPR**	**TNR**
Domain1	Domain2	0.9434	0.8190	0.8910	0.9512
Domain1	Domain3	0.9641	0.8993	0.9611	0.9607
Domain2	Domain1	0.8645	0.7425	0.9958	0.8360
Domain2	Domain3	0.8256	0.5947	0.9923	0.8047
Domain3	Domain1	0.9740	0.9249	0.8903	0.9942
Domain3	Domain2	0.9670	0.8582	0.7972	0.9942
Average		0.9231	0.8064	0.9213	0.9235

**Table 4:** Results on ultrasound thyroid nodule datasets with PAN (43) +AUG.

**Source**	**Target**	**PA**	**DSC**	**TPR**	**TNR**
Domain1	Domain2	0.9270	0.8136	0.9463	0.9219
Domain1	Domain3	0.9449	0.8557	0.9663	0.9382
Domain2	Domain1	0.8915	0.7720	0.9931	0.8692
Domain2	Domain3	0.8378	0.6156	0.9900	0.8189
Domain3	Domain1	0.9691	0.9076	0.8661	0.9939
Domain3	Domain2	0.9618	0.8341	0.7633	0.9946
Average		0.9220	0.7998	0.9209	0.9228

**Table 5:** Results on ultrasound thyroid nodule datasets with TRFEplus (38) + AUG.

**Source**	**Target**	**PA**	**DSC**	**TPR**	**TNR**
Domain1	Domain2	0.9597	0.8633	0.9451	0.9602
Domain1	Domain3	0.9637	0.8609	0.8588	0.9783
Domain2	Domain1	0.9473	0.8408	0.7743	0.9855
Domain2	Domain3	0.9431	0.7641	0.7242	0.9779
Domain3	Domain1	0.9570	0.8756	0.8304	0.9874
Domain3	Domain2	0.9430	0.8007	0.8580	0.9611
Average		0.9523	0.8342	0.8318	0.9750

**Table 6:** esults on ultrasound thyroid nodule datasets with SegM + AUG.

**Source**	**Target**	**PA**	**DSC**	**TPR**	**TNR**
Domain1	Domain2	0.9518	0.8443	0.9109	0.9566
Domain1	Domain3	0.9749	0.9105	0.9482	0.9773
Domain2	Domain1	0.8747	0.7548	0.9939	0.8486
Domain2	Domain3	0.8260	0.5932	0.9921	0.8054
Domain3	Domain1	0.9714	0.9155	0.8726	0.9955
Domain3	Domain2	0.9656	0.8548	0.8075	0.9910
Average		0.9274	0.8122	0.9209	0.9291

**Table 7:** Results on ultrasound thyroid nodule datasets with ours.

**Source**	**Target**	**PA**	**DSC**	**TPR**	**TNR**
Domain1	Domain2	0.9730	0.8947	0.9147	0.9839
Domain1	Domain3	0.9744	0.9063	0.9573	0.9795
Domain2	Domain1	0.9676	0.9106	0.9558	0.9724
Domain2	Domain3	0.9394	0.7778	0.9691	0.9408
Domain3	Domain1	0.9614	0.8949	0.8243	0.9977
Domain3	Domain2	0.9576	0.8392	0.8132	0.9899
Average		**0.9622**	**0.8706**	0.9057	**0.9774**

Bold values mean that the value is the best among all the comparison methods and our method.

**Figure 4:**
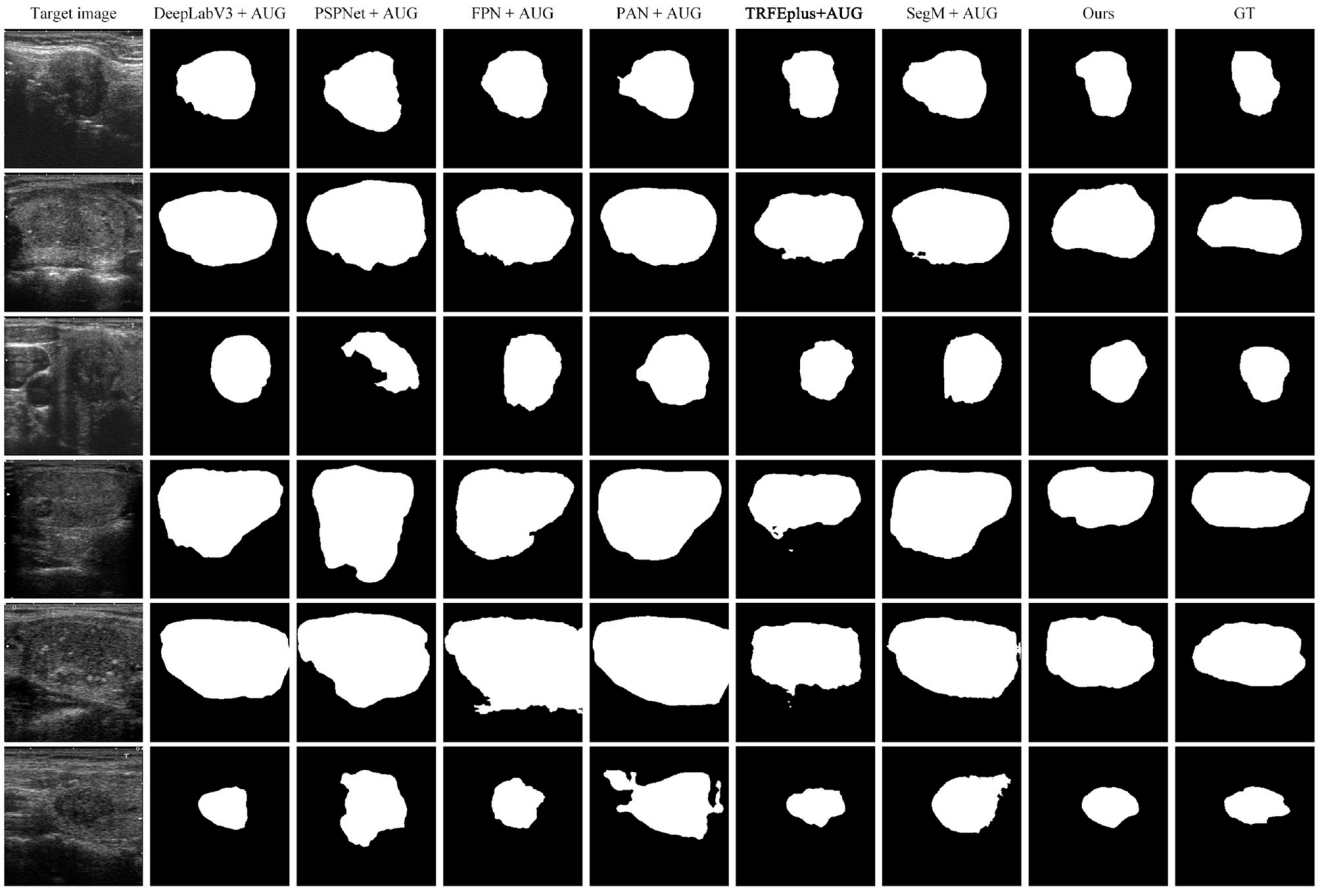
Comparison of cross-domain segmentation results on target domain. From left to right: target image, DeepLabV3+AUG, PSPNet+AUG, FPN+AUG, PAN+AUG, TRFEpuls+AUG, SegM+AUG, Ours, GT.

We further show the qualitative comparison of the methods in Figure 4. As can be seen, while other methods either fail to segment the nodules or over-segment a large portion of nodules, our method generates more accurate segmentation results.

### 3.3. Ablation study

To verify the advancement of our medical image translation module and the effectiveness of the consistency loss, we carry out a series of ablation experiments as follows: (a) **w/o translation module:** disabling the whole image translation module during training. (b) **w/o feature-level GAN:** disabling feature-level adversarial loss during training. (c) **w/o consistency loss:** disabling consistency loss during training; (d) **w/o DA:** only segmentation module.

The results of our ablation experiments are demonstrated in Table 8. From the results, we can summarize the following conclusions. (a) In the absence of translation module and feature-level GAN, *DSC* drops by 0.0459 and 0.0406, respectively, which proves that they play a certain role in improving the segmentation results and are of equal importance. (b) In the absence of consistency loss, *DSC* drops sharply by 0.18, which indicates that the segmentation consistency loss plays a decisive role in our network's performance. (c) When we only use our segmentation module to complete cross-domain tasks, the effect is not satisfactory, namely 0.6141 in *DSC*. It illustrates the effectiveness of our domain adaptation framework. In conclusion, our ablation experiments indicate that the proposed medical image translation module, the consistency loss and closing in the feature space are helpful to close the domain gap between source data and target data.

**Table 8:** Ablation study on various constraints.

**Ablation (w/o)**	**Translation module**	**Feature-level GAN**	**Consistency loss**	**DA**	**Ours**
**PA**	**0.9632**	**0.9644**	**0.9163**	**0.8613**	**0.9730**
DSC	0.8488	0.8541	0.7069	0.6141	**0.8947**

Bold value means that the value is the best in the ablation study.

## 4. Conclusion

In this paper, we have presented a domain adaptation method for medical image segmentation. In order to alleviate the domain shift problem caused by the difference in data styles, we propose to bridge the domain gap between multi-site medical data on both the pixel and feature level. Meanwhile, we introduce two symmetrical hybrid-attention segmentation modules to segment the source domain data and target domain data, respectively. Besides, we construct the segmentation consistency loss to guarantee the model stability. Experimental results on Ultrasound Thyroid Nodules datasets show the remarkable generalization ability of our proposed method.

## Data availability statement

The private datasets presented in this article are not readily available due to policy. Requests to access the datasets should be directed to corresponding authors.

## Ethics statement

The studies involving human participants were reviewed and approved by Medical Ethics Committee, Zhongnan Hospital of Wuhan University. The patients/participants provided their written informed consent to participate in this study.

## Author contributions

WM, XL, CF, LZ, and MW: conceptualization, writing—original draft preparation, and writing—review and editing. WM, XL, and CF: methodology and investigation. WM, XL, CF, and LZ: validation. WM, XL, CF, and MW: formal analysis. WM, CF, LZ, and MW: resources. WM, XL, and MW: data curation. WM and XL: visualization. CF, LZ, and MW: supervision and project administration. MW: funding acquisition. All authors have read and agreed to the published version of the manuscript.
